# Supplementary material for: Evaluation of a credit-bearing online administered happiness course on undergraduates’ mental well-being during the COVID-19 pandemic
Source: PLoS One. 2022 Feb 16;17(2):e0263514. doi: 10.1371/journal.pone.0263514 (PMC8849469; doi:10.1371/journal.pone.0263514)
Supplement: S1 File — (DOCX) [file pone.0263514.s001.docx]

**S1 File.** **Full results for hypotheses one and two when adjusting for Psychology Degree Programme.**

When enrolment in a psychology degree programme versus other degree programme was taken into account, the effects of our main analyses were maintained.

*SWEMWBS*

We again observed a significant interaction between group and timepoint (timepoint 1 to 2: β = -1.24, 95% CI: -1.89, -0.58, p < .001; timepoint 1 to 3: β = -0.89, 95% CI: -1.52, -0.25, p = 0.006). There was no difference in scores between those in a psychology programme and other programmes (β = -0.26, 95% CI: -0.89, 0.38, p = 0.434).

*GAD-7*

Results were comparable to our original analyses (timepoint 1 to 2: β = 1.18, 95% CI: 0.20, 2.16, p = 0.019; timepoint 1 to 3: β = 1.25, 95% CI: 0.30, 2.19, p = 0.010). Psychology degree was not associated with anxiety (β = -0.15, 95% CI: -1.14, 0.85, p = 0.770).

*SHS*

Effects were maintained; we observed a significant interaction with group and timepoints 1 to 2 (β = -0.18, 95% CI: -0.34, -0.02, p = 0.033) but no interaction effect at timepoints 1 to 3 (β = -0.11, 95% CI: -0.26, 0.05, p = 0.173). Psychology degree was not associated with SHS scores (β = 0.01, 95% CI: -0.24, 0.25, p = 0.949).

*Satisfaction and worry about academic performance*

Similar results were observed after taking into account degree programme; satisfaction or worry about academic performance did not differ by group (satisfaction: β = 0.01, 95% CI: -0.17, 0.20, p = 0.895; worry: β = 0.15, 95% CI: -0.09, 0.39, p = 0.220). Degree programme was not associated with satisfaction (β = -0.05, 95% CI: -0.24, 0.13, p = 0.589) or worry (β = -0.10, 95% CI: -0.34, 0.14, p = 0.408).
