# Supplementary material for: Evaluation of a credit-bearing online administered happiness course on undergraduates’ mental well-being during the COVID-19 pandemic
Source: PLoS One. 2022 Feb 16;17(2):e0263514. doi: 10.1371/journal.pone.0263514 (PMC8849469; doi:10.1371/journal.pone.0263514)
Supplement: S1 Table — (DOCX) [file pone.0263514.s002.docx]

**S1 Table. Mean change and standardised difference scores (Cohen’s *d_z_*) with 95% confidence intervals for SWEMWBS Metric Scores according to GRC response.**

|  | Time 1 to 2 | | | | Time 1 to 3 | | | |
| --- | --- | --- | --- | --- | --- | --- | --- | --- |
|  | Intervention (n = 145) | | Wait-List (n = 153) | | Intervention (n = 145) | | Wait-List (n = 189) | |
|  | Mean Change | *d_z_* | Mean Change | *d_z_* | Mean Change | *d_z_* | Mean Change | *d_z_* |
| GRC Response |  |  |  |  |  |  |  |  |
| A lot better | 2.17 (1.06, 3.27) | 0.79 (0.35, 1.23) | 0.40 (-1.31, 2.11) | 0.15 (-0.42, 0.71) | 2.08 (0.59, 3.58) | 0.62 (0.15, 1.07) | 1.18 (-0.68, 3.03) | 0.33 (-0.17, 0.81) |
| A little better | 0.83 (0.21, 1.44) | 0.35 (0.09, 0.61) | 0.21 (-0.42, 0.84) | 0.10 (-0.20, 0.40) | 1.05 (0.38, 1.71) | 0.48 (0.16, 0.79) | 0.20 (-0.60, 0.98) | 0.07 (-0.21, 0.36) |
| About the Same | -0.60 (-2.17, 0.98) | -0.15 (-0.54, 0.24) | -0.61 (-1.27, 0.06) | -0.31 (-0.64, 0.03) | -0.24 (-1.28, 0.79) | -0.08 (-0.40, 0.25) | -0.93 (-1.57, -0.29) | -0.45 (-0.77, -0.13) |
| A little worse | -1.07 (-2.11, -0.02) | -0.40 (-0.78, -0.01) | -1.94 (-2.73, -1.16) | -0.77 (-1.10, - 0.42) | -2.02 (-3.20, -0.85) | -0.64 (-1.03, -0.25) | -2.51 (-3.23, -1.80) | -0.93 (-1.24, -0.62) |
| A lot worse | -4.74 (-8.08, 1.40) | -1.76 (-3.19, -0.27) | -3.07 (-4.39, -1.74) | -1.08 (-1.63, -0.52) | -4.03 (-5.72, -2.33) | -1.44 (-2.21, -0.64) | -2.80 (-3.77, -1.83) | -1.20 (-1.70, -0.67) |
