# Supplementary material for: Evaluation of a credit-bearing online administered happiness course on undergraduates’ mental well-being during the COVID-19 pandemic
Source: PLoS One. 2022 Feb 16;17(2):e0263514. doi: 10.1371/journal.pone.0263514 (PMC8849469; doi:10.1371/journal.pone.0263514)
Supplement: S2 Table — (DOCX) [file pone.0263514.s003.docx]

**S2 Table. Agreement with items relating to positive perceptions of university and interest in activities that can increase well-being in the intervention group at timepoint one.**

| Item | N (%) |
| --- | --- |
| *I feel positive about being at University* |  |
| Strongly Agree | 18 (10.84) |
| Agree | 89 (53.61) |
| Somewhat Agree | 44 (26.51) |
| Neither Agree nor Disagree | 3 (1.81) |
| Somewhat disagree | 8 (4.82) |
| Disagree | 4 (2.41) |
| Strongly Disagree | 0 (0) |
| *I am actively interested in trying activities that could increase my well-being* | |
| Strongly Agree | 55 (33.13) |
| Agree | 55 (33.13) |
| Somewhat Agree | 41 (24.70) |
| Neither Agree nor Disagree | 9 (5.42) |
| Somewhat disagree | 6 (3.61) |
| Disagree | 0 (0) |
| Strongly Disagree | 0 (0) |
